# Umbilical cord blood CD34^+^ cells administration improved neurobehavioral status and alleviated brain injury in a mouse model of cerebral palsy

**DOI:** 10.1007/s00381-021-05068-0

**Published:** 2021-02-09

**Authors:** Yanqun Chang, Shouheng Lin, Yongsheng Li, Song Liu, Tianbao Ma, Wei Wei

**Affiliations:** 1 Department of Medical Rehabilitation, Guangdong Women and Children Hospital, Guangzhou, China; 2 Key Laboratory of Regenerative Biology, Guangzhou Institutes of Biomedicine and Health, Chinese Academy of Sciences, Guangzhou, China; 3 Guangdong Cord Blood Bank, Guangzhou, China; 4 Guangzhou Municipality Tianhe Nuoya Bio-engineering Co., Ltd., Guangzhou, China; 5 Guangzhou Reborn Health Management Consultation Co., Ltd., Guangzhou, China

**Keywords:** Umbilical cord blood, Brain injury, Cerebral palsy, Mouse model

## Abstract

**Purpose:**

Cerebral palsy (CP) is the most common neuromuscular disease in children, and currently, there is no cure. Several studies have reported the benefits of umbilical cord blood (UCB) cell treatment for CP. However, these studies either examined the effects of UCB cell fraction with a short experimental period or used neonatal rat models for a long-term study which displayed an insufficient immunological reaction and clearance of human stem cells. Here, we developed a CP model by hypoxia-ischemic injury (HI) using immunodeficient mice and examined the effects of human UCB CD34^**+**^ hematopoietic stem cells (HSCs) on CP therapy over a period of 8 weeks.

**Methods:**

Sixty postnatal day-9 (P9) mouse pups were randomly divided into 4 groups (*n* = 15/group) as follows: (1) sham operation (control group), (2) HI-induced CP model, (3) CP model with CD34^**+**^ HSC transplantation, and (4) CP model with CD34^-^ cell transplantation. Eight weeks after insult, the sensorimotor performance was analyzed by rotarod treadmill, gait dynamic, and open field assays. The pathological changes in brain tissue of mice were determined by HE staining, Nissl staining, and MBP immunohistochemistry of the hippocampus in the mice.

**Results:**

HI brain injury in mice pups resulted in significant behavioral deficits and loss of neurons. Both CD34^+^ HSCs and CD34^-^ cells improved the neurobehavioral statuses and alleviated the pathological brain injury. In comparison with CD34^-^ cells, the CD34^+^ HSC compartments were more effective.

**Conclusion:**

These findings indicate that CD34^+^ HSC transplantation was neuroprotective in neonatal mice and could be an effective therapy for CP.

## Introduction

Cerebral palsy (CP) is the most common physical disability in childhood. Children with CP exhibit a complex set of dysfunctions, including behavioral disorders, disturbances in sensation and perception, epilepsy, mental retardation, and language deficits[1]. Nowadays, many treatments have been developed for CP, which include diverse surgeries on muscles, tendons, bone, and nerves [2]; medications [3]; electrical stimulation, patterning, and conductive education [4]; and orthoses [5]. However, these treatments only provide symptomatic relief, and some may cause discomfort or severe side effects in the patients. Overall, CP is still incurable and remains an urgent topic in pediatric neurodevelopmental research.

Recently, cell therapy has attracted huge attention as a new treatment for CP. For treatments of many disorders, especially perinatal brain injury, there are many advantages in the use of umbilical cord blood (UCB) mononuclear cells, including the availability, high tolerance to HLA mismatch, and reduced graft versus host disease [6]. Several studies demonstrated that UCB cells could migrate to sites of injury within the brain in CP rats [7], reduced functional deficits [8], and protected against white matter injury [9]. The analysis indicated that UCB cells were more effective than any other tested cell types at treating children with CP, with a significant intervention effect in a short-term (6 months) follow-up[10]. However, UCB cells are a variable mix of stem and progenitor cells, including hematopoietic stem cells (HSCs), endothelial progenitor cells (EPCs), and mesenchymal stem cells (MSCs). It is still not clear how each of these cell types individually contributes to neurorepair. It is difficult to quantify the potency and efficacy of UCB cells in specific clinical circumstances. Therefore, it is critical to develop new specific single cell type therapies.

HSCs defined as CD34^**+**^ cells have been shown to significantly improve behavioral outcomes and increase neurogenesis after stroke in both adult and neonatal models[7, 11]. This study mainly aimed to examine the capability of CD34^+^ HSCs in brain injury treatment in mice with CP during a long-term period (8 weeks) in mice with CP, which approaches to maturity 8 weeks after birth, when the various organs of the bodies are mature and all functions of them are basically determined.

## Methods

### Isolation of human CD34^+^ cells

All human UCB samples were obtained from Guangdong Cord Blood Bank. Written informed consent was obtained from each donor. The use of these samples in this study was approved by the Committee for the Ethical Review of Research Involving Human Subjects at Guangdong Cord Blood Bank. CD34^**+**^ cells were collected using human CD34 MicroBead Kit UltraPure (Miltenyi Biotec, 130-100-453). The purity was > 90%, and the viability of the cells was > 95%. The remaining fraction was identified as CD34^-^ cells.

### Animals and surgery

Animal experiments were performed in the Laboratory Animal Center of the Guangzhou Institutes of Biomedicine and Health (GIBH), and all animal procedures were approved by the Animal Welfare Committee of GIBH. NOD-SCID-IL2Rg−/− (NSI) mice were derived at the GIBH. Mice were housed in specific pathogen-free cages and provided with autoclaved food and water. Protocols were approved by the relevant Institutional Animal Care and Use Committee (IACUC).

Sixty postnatal day-9 (P9) mouse pups were prepared for the experiments. P8–12 mice were considered comparable to human full-term (P0) neonates in regard to brain maturation[12]. Pups were randomly divided into 4 groups (*n* = 15/group) as follows: (1) sham operation (control group), (2) hypoxic-ischemic injury (HI)-induced CP model, (3) CP model with CD34^**+**^ HSC transplantation, and (4) CP model with CD34^-^ cell transplantation. Mice were anesthetized with 3.5% isoflurane for induction and 1.5% for maintenance. The left common carotid artery was dissected and permanently ligated with a prolene suture. The skin incision was then sutured. Pups were returned to their mothers for 1 h and then placed in a plexiglass hypoxia chamber at 37 °C. A nitrogen-oxygen mixture of 8.0 % oxygen was injected into the chamber at 1.0–2.0 L/min for 0.5 hour. The pups were then returned to their cages and fed by their mothers. Sham-operated pups only underwent a left common carotid artery isolation without ligation or hypoxia.

### Intracerebral transplantation

12 hours after modeling, mice were fixed onto a stereo locator, followed by disinfection of the scalp, sagittal incision, and drilling under aseptic operation. The transplantation site was located on a location of ( AP: −1 mm, ML: −1 mm, DV: −2 mm, 1 mm after the bregma, 1 mm left to the bregma, and 2 mm deep). The needle was slowly inserted 1.5 mm vertically by using a microsyringe, and 2 μl of cell mixture were injected (about 1×10^5^). The injection process took more than 5 min and the needle was kept in place for 5 min and then was gradually pulled out for 5 min. The scalp was sutured and mice were returned to their cages after warming and awakening.

### Accelerating rotarod

The rotarod apparatus was composed of a horizontal rod, 3 cm in diameter, separated by opaque plastic dividers in order to accommodate up to 5 mice per trial (Ugo Basile, 47600). The rotarod accelerated from 4 to 40 rpm over 5 min. The latency to fall from the rod was recorded for three consecutive sessions on test day.

### Gait dynamic assay

Gait dynamic assays were performed using the DigiGait imaging system along with DigiGait 10.0 analysis software (Mouse Specifics). This imaging system has been described in greater detail elsewhere [13]. The DigiGait apparatus consists of a clear plastic treadmill with a high-speed under-mounted digital camera (Basler Technologies Inc.) used for imaging paw prints. Images were collected at a rate of 140 frames/s and stored as audio video interleaved (AVI) files for later analysis. Image analysis software digitally encoded animal paw area and position relative to the tread-belt. Each paw of the animal was treated as a unique signature such that later analysis of foot movement could be performed on separate limbs. An average of 10 sequential strides per paw was collected from each mouse. This number of strides had been validated as being sufficient to analyze treadmill walking behavior in mice[14]. All settings, i.e., camera, lighting, and belt speed, were optimized before experimental testing.

Mice were locked into a clear chamber which was placed on the treadmill serving as the floor. The mice were allowed to explore the habitation for 1 min with slow treadmill activation (10 cm/s). On subsequent trials, animals were tasked with walking at a faster pace (20 cm/s).

### Open field

The activity of each mouse on the first day of habituation to the object recognition test apparatus was recorded as an open field task to study locomotor and exploratory activities[15]. Individual mice were placed in the square arena and allowed to explore the region for 10 min. The area was divided in 16 square zones, of which the four central squares were considered the central zone. The data were presented as average speed (cm/s), total activity (%), total distance travelled (cm), distance travelled in the center (%), and time spent in the center (%).

### Histological analyses and immunohistochemical staining

After neurobehavioral evaluation, mice were perfusion-fixed intracardially with 4% paraformaldehyde. The brain was removed and fixed in a 4% paraformaldehyde solution for 24 h; thereafter it was dehydrated, embedded, and sectioned continuously into 4-μm tissues sections for histological examination. The slices were placed in an incubator at 60 °C for 2 h and dewaxed, hydrated, and stained with hematoxylin for 5 min; differentiated with 1% hydrochloric acid alcohol; and immersed for 10 min. Afterward, the slices were stained with 1% water-soluble eosin, dehydrated with 75% alcohol for 3 min, 85% alcohol 3 min, and 95% alcohol for 3 min, followed by dehydration, clearance, and sealing. The histopathological condition of brain tissues was observed by the histopathological examination and photographs.

Paraffin-embedded sections were deparaffinized and, after heat-mediated antigen retrieval, sealed by 5% goat serum and incubated with an antibody against MBP (Abcam, ab40390, 1:200) at 4 °C overnight. The sections were incubated with a peroxidase-labeled antibody at 37 °C for 20 min. The slides were then stained with DAB and counterstained with hematoxylin, followed by conventional dehydration, clearance, and sealing. All slides were imaged with a microscope (DMI6000B; Leica Microsystems). For each specimen, 4 fields were visualized to calculate the mean integrated optical density (IOD) of MBP protein expression. All the immunochemistry results were analyzed using Image-Pro Plus 6.0 software.

### Statistical analysis

Data analysis was performed using GraphPad Prism (GraphPad Software). A two-way repeated measures ANOVA was used to establish statistical significance. Differences were considered significant at *P* < 0.05. The results were expressed as the mean ± standard deviation (SD), unless otherwise noted.

## Results

### Effect of CD34^+^ HSC transplantation on sensorimotor performance in HI-induced mice

**Fig. 1:**
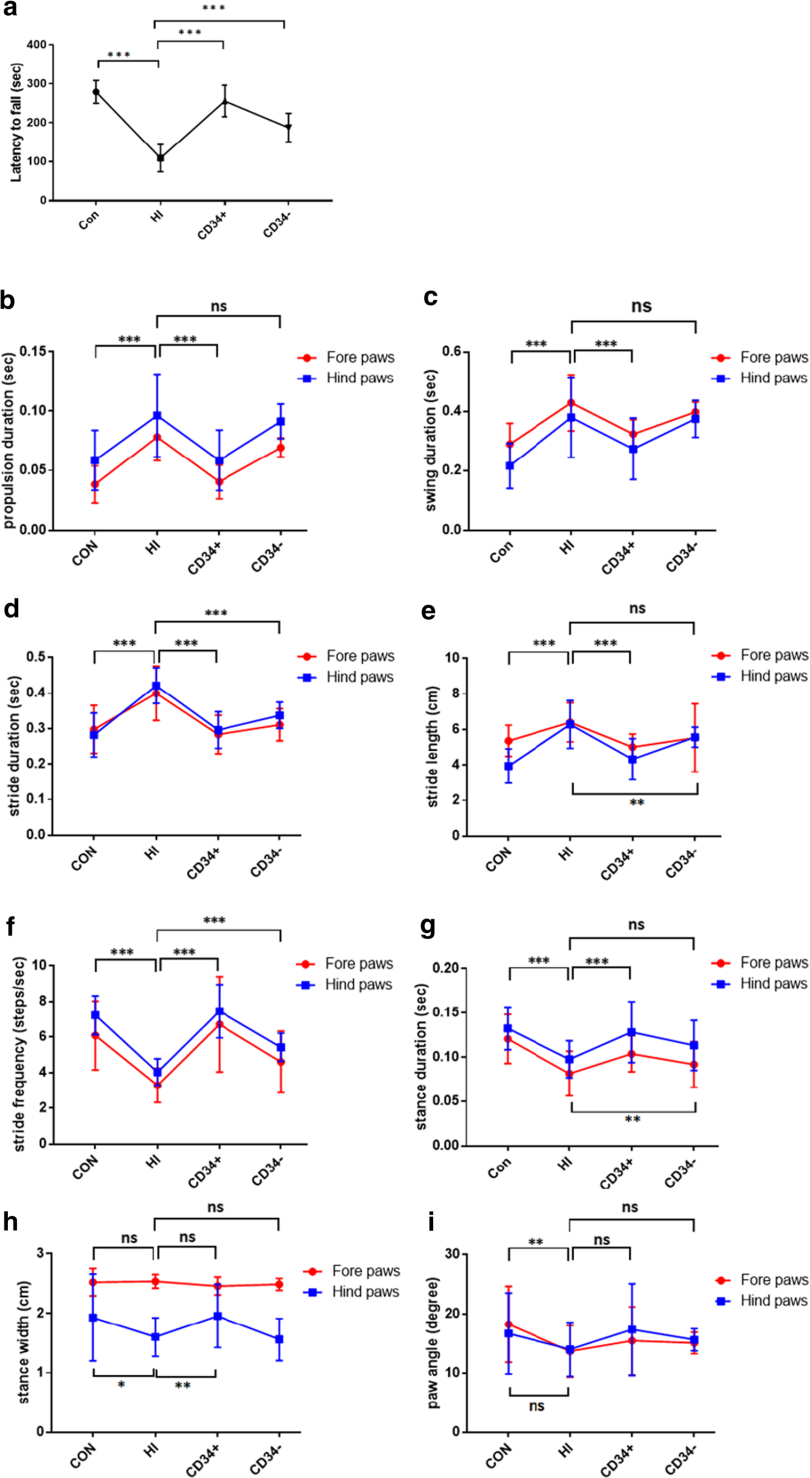
CD34^**+**^ HSC transplantation improved Rotarod and gait performance. (A) Latency to fall from an accelerated rotarod. Dynamic measurements of gait detected by the DigiGait apparatus: (B) propulsion duration (sec); (C) swing duration (sec); (D) stride duration (sec); (E) stride length (cm); (F) stride frequency (step/sec); (G) stance duration (sec); (H) stance width (cm); and (I) paw angle (degree). CON: sham operated; HI: hypoxic–ischemic injury; CD34^**+**^: HSC transplantation; CD34^-^: CD34^-^ cell transplantation. *p < 0.05, **p < 0.01, and ***p < 0.001. Error bars represent ± SD

Eight weeks after insult, the sensorimotor performance was analyzed by a rotarod treadmill. A statistically significant difference was observed between the HI-induced group and the stem cell therapy groups. Compared with the sham-surgery group, the performance was significantly impaired in the HI-induced group, and it was rescued in mice transplanted with CD34^**+**^ HSCs and CD34^-^ cells. It showed a better curative effect on the CD34^+^ HSC group than the CD34^-^ cell group (Fig. 1a).

### CD34^+^ HSC transplantation improves ataxic perturbation in HI-induced mice

All gait dynamic assays were performed using the DigiGait imaging system along with DigiGait 10 analysis software to detect changes in movement patterns.

Propulsion duration indicated a prolonged extension of both the fore and hind paws in HI-induced mice[16]. CD34^**+**^ HSC therapy restored the normal propulsion duration, whereas CD34^-^ cell treatment had no effect (Fig. 1b).

Swing duration is the time interval between the end of the propulsion phase and the beginning of the braking phase[16]. The results suggested that HI-induced mice had a longer swing duration than the control group. CD34^**+**^ HSC but not CD34^-^ cell had efficacy to improve the symptoms (Fig. 1c).

Stride duration is defined as the time from the beginning of one stance slope to the next [16]. Fore and hind paws performance exhibited significant effect of HI. CD34^**+**^ HSC treatment recovered normal duration in HI-induced mice and CD34^-^ cell treatment showed moderate efficacy (Fig. 1d). Furthermore, HI-induced mice exhibited a significant longer stride length and lower stride frequency, indicating a longer pace. Therapy with stem cells, especially CD34^**+**^ HSCs, improved motor performance (Fig. 1e-f).

Stance duration is calculated as the total time of both the braking and propulsion phases of the stance, while the paw was in contact with the belt [16]. HI-induced mice showed reduced stance time during walking. CD34^**+**^ HSC treatment regained stance duration in both fore and hind paw performance, while CD34^-^ cell treatment exhibited moderate improvement (Fig. 1g).

The stance width measured the distance between the centroids of the left and right paws[16]. HI resulted in a decreased stance width in the hind paws, indicating an unbalanced posture. CD34^**+**^ HSC treatment recovered the impair performance, but CD34^-^ cell treatment showed no improvement (Fig. 1h). No effect was detected in fore paw measurements.

Paw angle indicated the total angle of the left and right paws[16]. Only fore angle was significantly decreased in HI-induced mice, and no improvement was showed after stem cell therapy (Figure 1I).

### CD34^+^ HSC treatment ameliorates HI-induced exploratory behavior and locomotion impairment

**Fig. 2:**
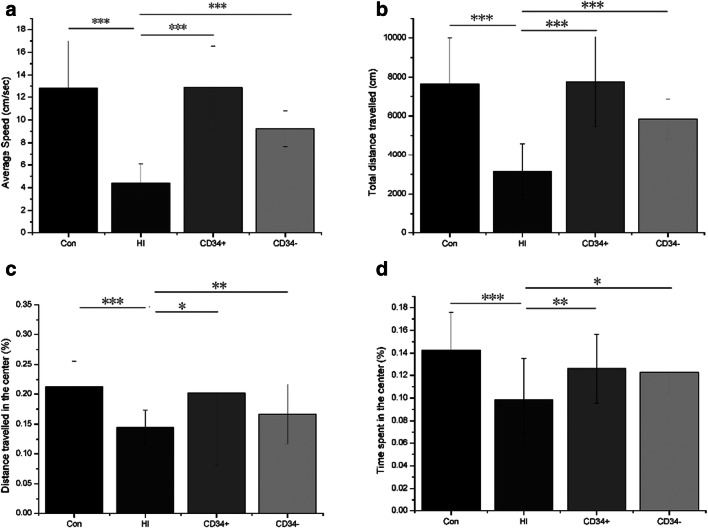
Locomotion and activity in open field test. (A) Average speed (cm/sec); (B) total distance travelled (cm); (C) distance travelled in the center (%); and (D) time spent in the center (%). Control: sham operated; HI: hypoxic–ischemic injury; CD34^**+**^: HSC transplantation; CD34^-^: CD34^-^ cells transplantation. *p < 0.05, **p < 0.01, and ***p < 0.001. Error bars represent ± SD

An open field evaluation was performed to confirm effects of HI or stem cell treatment on general exploratory behavior or locomotion of mice. Mice showed reduced speed and distance travelled in HI-induced group at 8 weeks after insult. Compared with HI-induced mice, CD34^**+**^ HSC or CD34^-^ cell treatment increased the locomotive speed and distance, indicating that HI impaired general exploratory and locomotor activities, stem cell therapy restored this impairment, while CD34^**+**^ HSCs had greater efficacy than CD34^-^ cells (Fig. 2a-b). Furthermore, the distance travelled and time spent in the center of the arena were decreased in HI-induced mice, indicating that anxiety behavior was induced by the HI procedure. Also, CD34^**+**^ HSC treatment had greater efficacy than CD34^-^ cells as a stem cell therapy (Figure 2C-D).

### CD34^+^ HSC treatment alleviates brain tissue injury in HI-induced mice

**Fig. 3:**
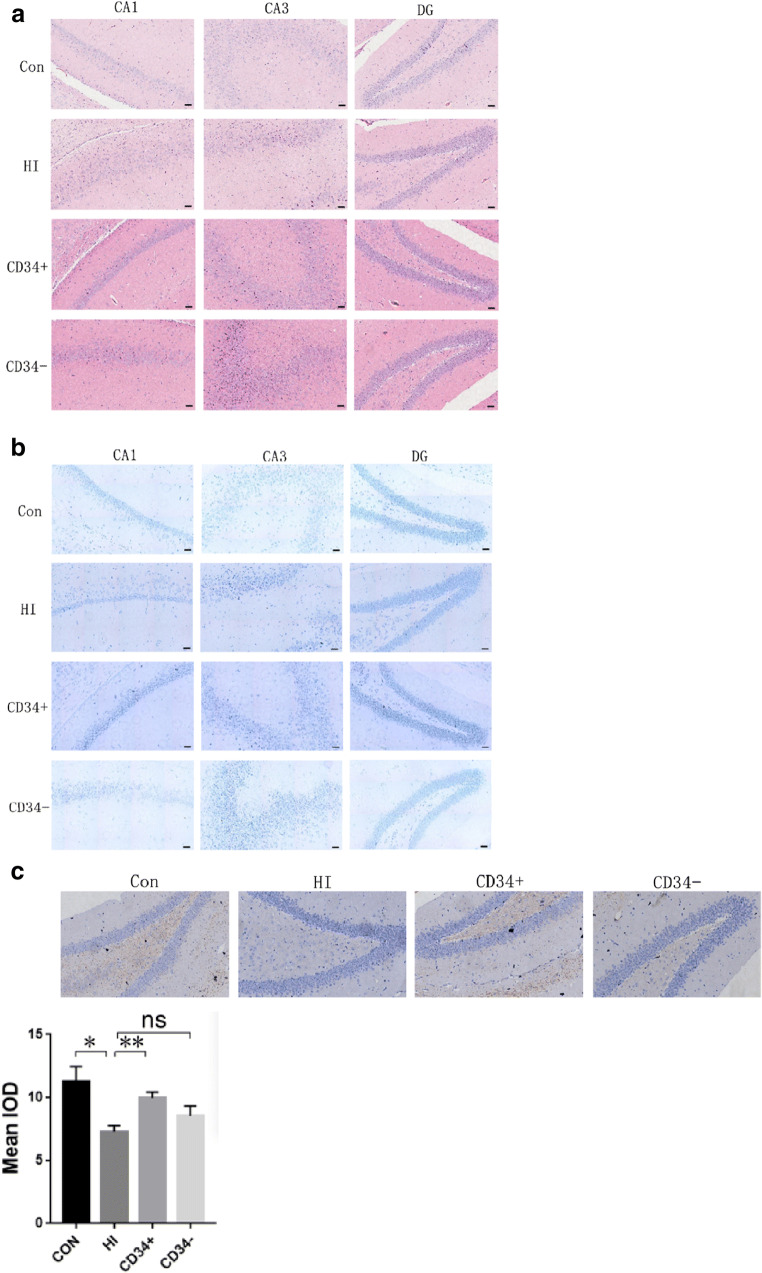
Pathological changes in brain tissue of mice. (A) Pathological observation of hippocampus in mice by HE staining. (B) Nissl stain of hippocampus in mice. (C) MBP immunohistochemistry of hippocampus in the mice, Strong MBP positive staining was observed in sham operated mice brain, staining was very weak at the same area in the HI-induced mice brain, strong positive staining for MBP was observed in HSCs treated mice brain, MBP staining in CD34^-^ cell group was generally weaker than sham and CD34^**+**^ groups. Con: sham operated; HI: hypoxic–ischemic injury; CD34^**+**^: HSC transplantation; CD34^-^: CD34^-^ cell transplantation. Staining intensity were showed as mean IOD (4 random sights/sample), data are presented as the mean ± SD. One-way ANOVA; ns: no significant difference; *p < 0.05, **p < 0.01, and ***p < 0.001

HE staining results showed that the cells of the hippocampus were arranged orderly and the shape was normal in the sham group. In the HI group, the cells in the hippocampus were swollen, denatured, and disorganized. In the CD34^**+**^ HSC treatment group, the cell morphology in the hippocampus tended to be normal. In the CD34^-^ cell treatment group, cell morphology was improved, and the tissue structure in the hippocampus was alleviated (Fig. 3a). Nissl staining results indicated that neuronal degeneration has been found to be prominent in CA1 and CA3 regions with HI. There was no noticeable neuronal loss in mice with CD34^**+**^ HSC administration, and an incomplete restoration of neuronal numbers was observed when treated with CD34^-^ cells (Fig. 3b). Little staining for MBP was found in HI-induced mice brain, while in both sham and CD34^**+**^ HSC groups, strong positive staining for MBP was observed in the hippocampus. MBP staining in CD34^-^ cell group was generally weaker than sham and CD34^**+**^ HSC groups (Fig. 3c). The results suggested that CD34^**+**^ HSC transplantation could attenuate brain tissue injury in HI-induced mice.

## Discussion

In recent years, numerous methods have been developed for CP therapy, including occupational and physical therapies[3, 17, 18]. Despite the great advances achieved in obstetric and neonatal care, CP patients still have poor prognosis due to the lack of effective therapies. Thus, there is an urgent need to develop a treatment for this disease. In this present study, a mice model of CP with HI was established and used to examine the potential of HSCs in brain injury therapy over a long-term period.

First, we found that after HSC transplantation in CP mice, the neurobehavioral was improved. In the accelerating rotarod paradigm, HI-induced mice had less latency to fall, showing a deterioration in motor performance, which was improved after CD34^**+**^ HSC administration. The use of digital footprint analysis was a useful approach for quantifying gait dysfunction in mouse model. The dynamic assays showed a disparity in stride, stance, swing, and propulsion duration measurements between HI-induced CP mice and HSC-treated CP mice, indicating changes in movement patterns. CD34^**+**^ HSC treatment reverted these changes. Interestingly, we found that HI-induced mice may employ a different strategy to maintain balance. HI-induced mice decreased their hind paw stance width, which may be attributed to the need for increasing stability[19]. But HI-induced mice decreased their paw angle, and this behavior was not in line with stereotypical trial of human ataxia [20]. As an increase in paw angle would provide stability, the decreased paw angle may indicate imbalanced gait in HI-induced CP mice. However, in human, ataxia gait is defined as a decreased in stride length and stride frequency [21], and HI-induced mice showed increased in stride length. Humans are bipedal and the locomotion is more prone to instability. Thus these comparisons may be viewed in an appropriate context but not taken to be exact replications of the human condition.

In addition, these findings indicating that HI changed locomotive and exploratory behavior of mice is in line with a previous report that severe HI altered behavior in an open-field test [22]. But we also found anxiety-like behavior in HI-induced mice, which is inconsistent with a previous study[23]. Also, we found decrease immunostaining of MBP, which is produced by oligodendrocytes, in HI-induced mice brain. This is an indication that myelination in HI-induced mice brain might be impaired. Although the brain and neurobehavioral impairments mentioned above were restored after CD34^**+**^ HSC administration, we could not find CD34^**+**^ cells in mice brain, probably because of the long experimental period. We also observed that CD34^-^ cell treatment had moderate effect on repairing brain injury and improving behavior. It may suggest that other cells, such as MSCs and EPCs, are contributing to CP therapy. This is supported by previous studies[24–27]. However, MSCs were present in USC sample at a low frequency and number [28] and EPC even lower. With the same dosage, CD34^**+**^ HSCs had greater efficacy for CP therapy than CD34^-^ cells in this study. Thus, CD34^**+**^ HSCs may be the priority of stem cell therapy for CP treatment.

Recently, a handful of studies have assessed the efficacy of UCB cells in large animal models (rabbits and sheep) of neonatal brain injury[29–31]. But a major limitation to the large animal trials is they were conducted over a relatively short experimental period. In part it is due to the challenges of maintaining these animals in a neonatal intensive care setting over a prolonged period, and the financial concern of keeping large animals. Thus, these studies may not to meet the crucial need for long-term data. Furthermore, both autologous [32–34] and allogeneic [35–37] UCB cells have been proved to be therapeutically safe and effective in clinical trials. However, these clinical trials are all performed over a short-time period, and the long-term effects of UCB cells administration, especially on the behavioral outcomes, have not been extensively studied [38]. Other studies have used human cells in rat models for long-term data [39, 40], but they are still insufficient for an increased risk of immunological reaction and clearance of stem cells. For these reasons, we established a CP model with immunodeficient mice to minimize the immunological rejection of human cells. We also conducted the experiment over a relatively long experimental period to confirm the improvement after the mice reached adulthood.

In this study, a single dose of CD34^+^ HSCs improved long-term behavioral outcomes and restored neuronal numbers. The result was roughly consistent with the study conducted by Penny TR et al. (Penny TR et al. 2020) [41] which administrated SD rats multiple doses of UCB and modulated pathological evidence of long-term brain injury better than the study conducted by Penny TR et al. (Penny TR et al. 2019) [42] which administrated SD rats by a single dose of UCB. According to the above comparisons, we speculated that CD34^+^ HSCs in UCB could be the main effector cell population in the treatment of CP. And we supposed that common SD rats should resist human cells more intensively than the immunodeficient mice. This probably implies human UCB cells, especially CD 34^+^ HSC, should have a long-term effectiveness in clinical trial for CP.

In conclusion, this present study demonstrated that CD34^**+**^ HSCs ameliorated brain injury and improved neurobehavior in a hypoxic-ischemic mice model. This study would facilitate the development of CP therapy. However, this study was only conducted in animal models, prospective future studies would be needed to demonstrate the safety and the efficacy of CD34^+^ HSCs and above all to understand the correct doses of these cells to be administered in the different age groups and also in human neonate with cerebral palsy.
